# Extracellular arginine is required but the arginine transporter CAT3 (*Slc7a3*) is dispensable for mouse normal and malignant hematopoiesis

**DOI:** 10.1038/s41598-022-24554-2

**Published:** 2022-12-17

**Authors:** Yuhan Yan, Chao Chen, Zhiguo Li, Jing Zhang, Narin Park, Cheng-Kui Qu

**Affiliations:** Department of Pediatrics, Aflac Cancer and Blood Disorders Center, Winship Cancer Institute, Children’s Healthcare of Atlanta, Emory University School of Medicine, 1760 Haygood Drive NE, HSRB E302, Atlanta, GA 30322 USA

**Keywords:** Haematopoietic stem cells, Haematopoiesis

## Abstract

Amino acid-mediated metabolism is one of the key catabolic and anabolic processes involved in diverse cellular functions. However, the role of the semi-essential amino acid arginine in normal and malignant hematopoietic cell development is poorly understood. Here we report that a continuous supply of exogenous arginine is required for the maintenance/function of normal hematopoietic stem cells (HSCs). Surprisingly, knockout of *Slc7a3* (CAT3), a major L-arginine transporter, does not affect HSCs in steady-state or under stress. Although *Slc7a3* is highly expressed in naïve and activated CD8 T cells, neither T cell development nor activation/proliferation is impacted by *Slc7a3* depletion. Furthermore, the *Slc7a3* deletion does not attenuate leukemia development driven by *Pten* loss or the oncogenic *Ptpn11*^*E76K*^ mutation. Arginine uptake assays reveal that L-arginine uptake is not disrupted in *Slc7a3* knockout cells. These data suggest that extracellular arginine is critically important for HSCs, but CAT3 is dispensable for normal hematopoiesis and leukemogenesis.

## Introduction

Arginine is one of the most versatile amino acids with many metabolic and regulatory roles, serving as a proteogenic amino acid as well as a precursor for critical molecules, such as nitric oxide, creatine, polyamines, nucleotides, glutamate, etc., that have vital roles in cellular functions^1^. In addition, by binding to CASTOR (arginine sensor), arginine stimulates the activation of mTORC1^2–4^, a mast cell growth controller that integrates diverse environmental inputs to coordinate various anabolic and catabolic processes in cells. While some amino acids have been shown to be important for the maintenance or function of hematopoietic stem cells (HSCs)^5,6^, which are highly responsive to cell signaling and metabolic regulation during self-renewal and differentiation into mature blood cell lineages, little is known about the role of arginine for HSCs.

Arginine is a semi-essential amino acid since endogenous arginine produced from citrulline or breakdown of proteins is not sufficient for biosynthesis and signaling functions in certain types of cells or under conditions of high demand. Dietary arginine is thus indispensable to meeting metabolic demands^1^. Furthermore, arginine is implicated in cancer development and progression. Cancer cells consume significantly more arginine compared to their normal counterparts due to robust biosynthesis and the downregulation of argininosuccinate synthetase (ASS1), a rate-limiting enzyme in arginine synthesis, in a range of hematological and solid tumors^7^. As such, arginine starvation/degradation has emerged as a potential therapy for several types of cancers^8–13^. However, the role of arginine and its metabolites in various normal or malignant cells remains incompletely understood.

The availability of arginine for metabolic functions and other arginine-relevant biological processes is largely determined by the uptake of extracellular arginine via specialized transporters on the plasma membrane, cationic amino acid transporters (CATs)^14,15^. CAT3, encoded by *Slc7a3* on the X chromosome, is primarily found in developing tissues of embryos, the adult thymus, and the brain^16–18^. This specific arginine transporter caught our attention because our previous gene expression profiling analyses revealed that the expression of *Slc7a3* (CAT3), but not *Slc7a1* (CAT1) or *Slc7a2* (CAT2), was upregulated by 6.6 fold in the mitochondrial phosphatidylinositol phosphate phosphatase *Ptpmt1* knockout HSCs, in which mitochondrial aerobic metabolism was suppressed^19,20^. This adaptive response of *Slc7a3* expression in *Ptpmt1* knockout HSCs raised the possibility that CAT3 may play an important role in HSCs. We therefore decided to generate *Slc7a3* knockout mice and determine the role of arginine and *Slc7a3* in hematopoietic cell development and leukemogenesis.

## Results

### Exogenous arginine is required but the arginine transporter CAT3 is dispensable for HSC maintenance and function

**Figure 1:**
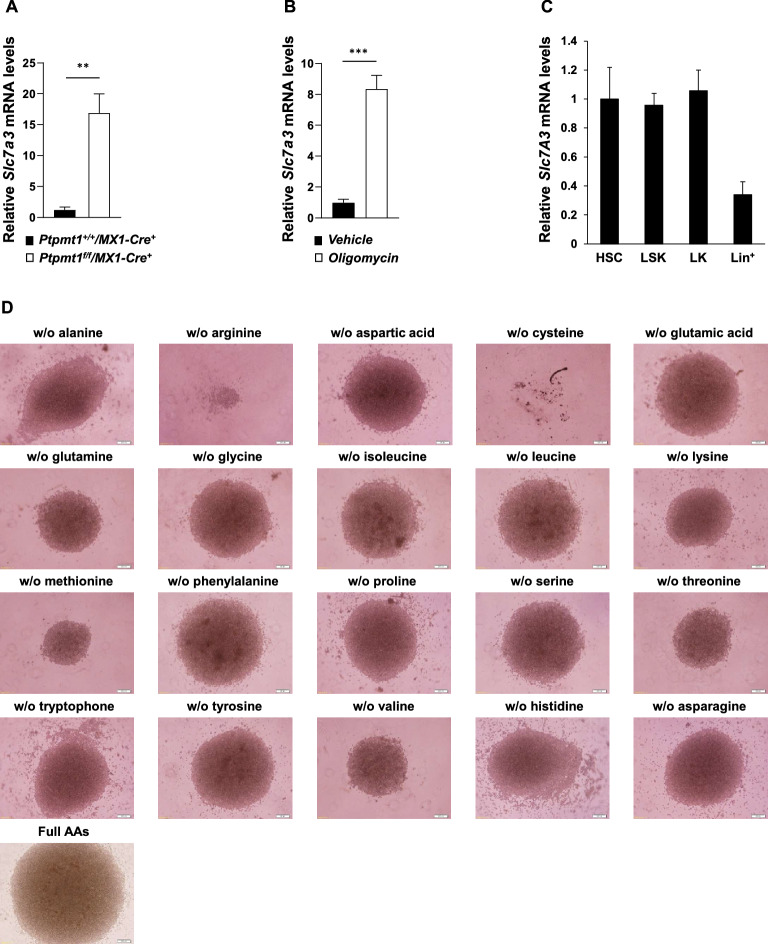
*Slc7a3* is highly upregulated in response to defective mitochondrial metabolism, and exogenous arginine is required for the maintenance and/or function of HSCs. (**A**) LSK (Lin^-^Sca-1^+^c-Kit^+^) cells were sorted from the BM isolated from pI-pC-administered *Ptpmt1*^*f/f*^*/MX1-Cre*^+^ and *Ptpmt1*^+*/*+^*/MX1-Cre*^+^ mice (n = 3 mice/group). Total RNA was extracted. *Slc7a3* mRNA levels were determined by qRT-PCR. (**B**) Lin^−^ cells purified from the BM cells isolated from WT C57BL/6 J mice (n = 3 mice/group) were treated with oligomycin (1 µM) or vehicle for 24 h. *Slc7a3* mRNA levels were determined as above. (**C**) Total RNA was extracted from HSCs (Lin^−^Sca-1^+^c-Kit^+^CD150^+^CD48^−^), LSK (Lin^−^Sca-1^+^c-Kit^+^) cells, LK (Lin^−^Sca-1^-^c-Kit^+^) cells, and Lin^+^ cells sorted from healthy WT C57BL/6 mice (n = 3 mice/genotype). *Slc7a3* mRNA levels were determined by qRT-PCR. (**D**) LSK cells freshly sorted from WT C57BL/6 mice were cultured (500 cells/well) in Earle’s Balanced Salt Solution supplemented with 10% FBS, Minimum Essential Medium vitamins (1x), SCF (50 ng/ml), IL-3 (20 ng/ml), IL-6 (20 ng/ml), and a mixture of amino acids that lacked the indicated specific amino acid. Seven days later, cells in each well were photographed.

Our recent gene expression profiling showed that *Slc7a3* (CAT3), a major arginine transporter, was greatly upregulated in *Ptpmt1* knockout hematopoietic stem/progenitor cells in which mitochondrial aerobic metabolism was inhibited^20^. Quantitative RT-PCR (qRT-PCR) analyses verified that *Slc7a3* expression levels were increased by ~ 17-fold in *Ptpmt1* knockout Lin^−^Sca-1^+^c-Kit^+^ (LSK) cells (early progenitor cells enriched with HSCs) (Fig. 1A). This data suggests that arginine-mediated metabolism compensates for defective mitochondrial metabolism. Indeed, *Slc7a3* expression increased by ~ eightfold when wild-type (WT) lineage negative (Lin^−^) cells were treated with the mitochondrial inhibitor oligomycin (Fig. 1B). Given these observations, we sought to determine the potential role of arginine metabolism in hematopoiesis. We examined expression levels of *Slc7a3* in hematopoietic cells in different developmental stages. *Slc7a3* expression in HSCs, LSK cells, and myeloid progenitor LK (Lin^−^Sca-1^-^c-Kit^+^) cells was ~ 3 times higher than that in relatively mature Lin^+^ cells (Fig. 1C). Furthermore, we cultured purified LSK cells in the absence of specific single amino acids and evaluated cell responses. After 7 days of culture, essentially all cells died in the wells without cysteine due to the essential role of this amino acid in the maintenance of cellular redox homeostasis. Interestingly, the deprivation of arginine from the culture medium also resulted in a robust detrimental effect (Fig. 1D). Arginine-deprived cells failed to proliferate and/or differentiate, and this effect was even greater than that caused by the deprivation of the essential amino acids valine, leucine, and isoleucine. These results suggest that a continuous supply of exogenous arginine is required for the maintenance and/or function of hematopoietic stem/progenitor cells.

**Figure 2:**
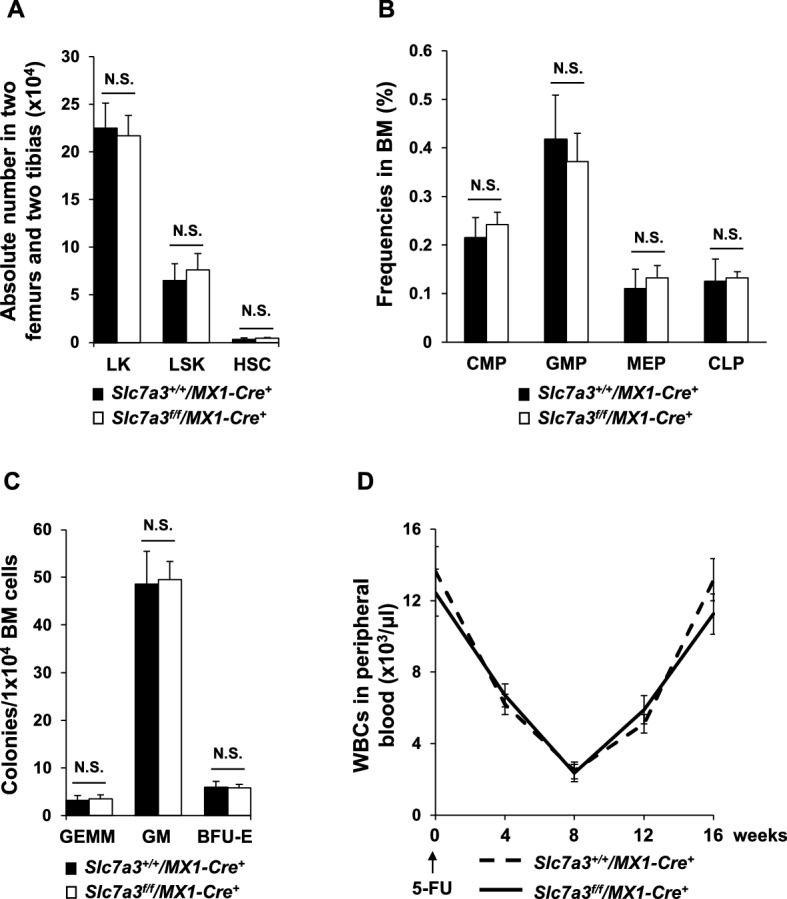
*Slc7a3* (CAT3) is dispensable for normal hematopoiesis at steady-state and in response to stress. (**A**-**C**) BM cells freshly harvested from *Slc7a3*^+*/*+^*/MX1-Cre*^+^ and *Slc7a3*^*f/f*^*/MX1-Cre*^+^ mice 5 weeks after pI-pC administration were assayed by FACS analyses to determine the absolute numbers of HSCs, LSK cells, and LK cells (**A**) (n = 5–6 mice/genotype) and the frequencies of CMPs, GMPs, MEPs, and CLPs (**B**) (n = 4–5 mice/genotype). BM cells (2 × 10^4^ cells) were assayed by CFU assays (n = 5–6 mice/genotype). Hematopoietic colonies (CFU-GEMM, CFU-GM, and BFU-E) were enumerated 10 days later. (**D**) Ten-week-old *Slc7a3*^+*/*+^*/MX1-Cre*^+^ mice and *Slc7a3*^*f/f*^*/MX1-Cre*^+^ littermates were administrated pI-pC. Five weeks later, the mice were treated with 5-fluorouracil (5-FU) (250 mg/kg body weight, i.p.) (n = 6 mice/genotype). White blood cell (WBC) counts in the peripheral blood were monitored.

To further determine the role of arginine-mediated signaling and metabolism in hematopoietic cell development, we created a *Slc7a3* conditional allele (*Slc7a3*^*flox*^) (Figures S1A and B) by gene targeting and generated inducible knockout mice (*Slc7a3*^*f/f*^*/MX1-Cre*^+^) by crossing *Slc7a3*^*f/*+^ mice with *MX1-Cre*^+^ transgenic mice that express the Cre DNA recombinase in pan-hematopoietic cells in response to polyinosinic-polycytidylic acid (pI-pC) administration^21^. pI-pC-induced deletion of *Slc7a3* in the bone marrow (BM) cells of these knockout mice was verified by qRT-PCR (Figure S1C). *Slc7a3* knockout mice did not display overt abnormalities during the 12-month follow-up. To assess the effects of *Slc7a3* abrogation on HSCs, we quantified phenotypic stem and progenitor cell populations in *Slc7a3*^*f/f*^*/MX1-Cre*^+^ mice 8 weeks after the deletion of *Slc7a3*. No significant changes in the absolute numbers of HSCs, LSK cells, and LK cells (myeloid biased progenitors) in the BM of *Slc7a3* knockout mice were observed (Fig. 2A). Moreover, frequencies of common myeloid progenitors (CMPs), granulocyte–macrophage progenitors (GMPs), megakaryocyte erythroid progenitors (MEPs), and common lymphoid progenitors (CLPs) were also comparable in *Slc7a3*^*f/f*^*/MX1-Cre*^+^ mice and *Slc7a3*^+*/*+^*/MX1-Cre*^+^ control littermates (Fig. 2B). Colony-forming unit (CFU) assays of BM cells showed similar numbers of functional progenitors, such as CFU-granulocytes, erythrocytes, macrophages, and megakaryocytes (CFU-GEMM), CFU-granulocytes and macrophages (CFU-GM), and burst forming unit-erythroid cells (BFU-E) in *Slc7a3*^*f/f*^*/MX1-Cre*^+^ and *Slc7a3*^+*/*+^*/MX1-Cre*^+^ mice (Fig. 2C). In addition, frequencies of myeloid (Mac-1^+^), B lymphoid (B220^+^), and T lymphoid (CD3^+^CD4^+^ and CD3^+^CD8^+^) cells in the BM were similar in *Slc7a3*^*f/f*^*/MX1-Cre*^+^ and *Slc7a3*^+*/*+^*/MX1-Cre*^+^ mice (Figure S2). Furthermore, we generated constitutive hematopoietic cell-specific *Slc7a3* knockout mice (*Slc7a3*^*f/f*^*/Vav1-Cre*^+^). These animals also appeared normal. Collectively, these data suggest that although *Slc7a3* is highly expressed in hematopoietic stem/progenitor cells, it is not required for steady-state hematopoiesis.

We next determined the role of *Slc7a3* in HSCs for hematopoietic regeneration in response to stress. We challenged *Slc7a3*^*f/f*^*/MX1-Cre*^+^ and *Slc7a3*^+*/*+^*/MX1-Cre*^+^ mice with 5-fluorouracil (5-FU), a cell-cycle-dependent myelotoxic agent that kills cells in proliferation, including cycling hematopoietic stem and progenitor cells. White blood cell (WBC) counts dropped quickly in all 5-FU-treated mice. Subsequent hematopoietic recovery from quiescent HSCs occurred in *Slc7a3*^*f/f*^*/MX1-Cre*^+^ mice as efficiently as that in *Slc7a3*^+*/*+^*/MX1-Cre*^+^ control littermates (Fig. 2D), suggesting that the deletion of *Slc7a3* produces no deleterious effects on the regenerative capabilities of long-term HSCs that require robust energetic and metabolic support.

### T cell development, activation, and proliferation are not disturbed in the absence of Slc7a3

**Figure 3:**
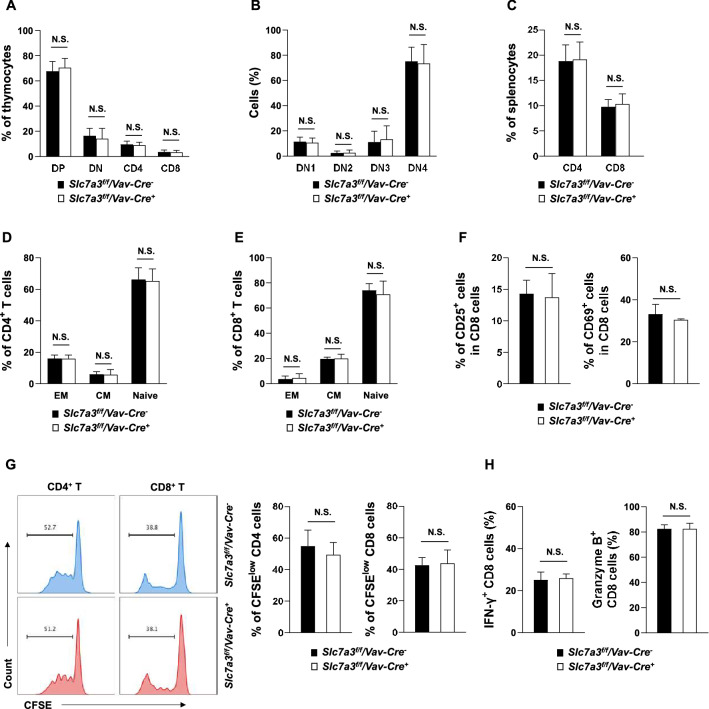
T cell development, activation, and proliferation are not disturbed by the deletion of *Slc7a3*. (**A**, **B**) Thymocytes freshly isolated from 8-week-old *Slc7a3*^*f/f*^*/Vav1-Cre*^+^ mice and *Slc7a3*^*f/f*^*/Vav1-Cre*^*−*^ littermates were stained with the indicated antibodies. Percentages of CD4 and CD8 double-negative (DN) cells, double-positive (DP), and CD4 and CD8 single-positive cells were quantified (n = 6 mice/genotype) (**A**). DN1 (CD44^+^CD25^−^), DN2 (CD44^+^CD25^+^), DN3 (CD44^−^CD25^+^), and DN4 (CD44^−^CD25^−^) cells in the DN population were further determined (n = 6 mice/genotype) (**B**). (**C**-**E**) Splenocytes freshly isolated from 8-week-old *Slc7a3*^*f/f*^*/Vav1-Cre*^+^ mice and *Slc7a3*^*f/f*^*/Vav1-Cre*^*−*^ littermates (n = 5 mice/genotype) were processed for FACS analyses. Frequencies of CD4^+^ and CD8^+^ T cells in the spleen were quantified (**C**). Percentages of T cells in various functional status (Effector memory T cells: CD44^+^CD62L^−^; Central memory T cells: CD44^+^CD62L^+^; Naïve T cells: CD44^−^CD62L^+^) in CD4^+^ (**D**) and CD8^+^ (**E**) populations were quantified. (**F**-**H**) CD8 T cells isolated from the spleen of *Slc7a3*^*f/f*^*/Vav1-Cre*^+^ and *Slc7a3*^*f/f*^*/Vav1-Cre*^*−*^ mice were labeled by carboxyfluorescein succinimidyl ester (CFSE) and stimulated with T cell activator anti-CD3/CD28 Dynabeads. Percentages of CD8 T cells expressing CD25 or CD69 were quantified by FACS 24 h after the stimulation (n = 5 mice/genotype) (**F**). Four days after anti-CD3/CD28 antibody stimulation, CFSE intensities in CD4 and CD8 T cells were assessed by FACS (n = 5 mice/genotype) (**G**). These cells were further stimulated with phorbol 12-myristate 13-acetate (PMA) (80 nM) in the presence of Golgistop (5 µg/ml) for 5 h. IFN-γ + and granzyme B + effector cells were determined by intracellular immunostaining followed by FACS analyses. Percentages of IFN-γ^+^ and granzyme B^+^ cells in CD8 T cell populations were determined (n = 5 mice/genotype) (**H**).

*Slc7a3* is highly expressed in the thymus^17^ in addition to the brain^18^. Indeed, *Slc7a3* has high expression levels in several CD8 T cell subsets in both mice and humans (Figure S3). To determine the potential role of *Slc7a3* in T cells, we examined T cells in the thymus and spleen in *Slc7a3*^*f/f*^*/Vav1-Cre*^+^ mice, but no defects in T cell development were observed. Percentages of double-positive (DP), double-negative (DN), CD4^+^, and CD8^+^ T cells in the thymus (Fig. 3A and B), and CD4^+^ and CD8^+^ T cells in the spleen (Fig. 3C) of the knockout mice were similar to those in control mice. Moreover, frequencies of naïve T cells (CD44^−^CD62L^+^), central memory T cells (CD44^+^CD62L^+^), and effector memory T cells (CD44^+^CD62L^−^) in both CD4 and CD8 T cell subpopulations were comparable in *Slc7a3* knockout and control mice (Fig. 3D and E), suggesting that the loss of *Slc7a3* has no impact on T cell homeostasis. In addition, *Slc7a3* knockout CD8 T cells could be efficiently activated, as evidenced by similar percentages of CD25^+^ and CD69^+^ cells derived from both knockout and control naive CD8 T cells (Fig. 3F). There was no difference in the proliferation of activated T cells between *Slc7a3* knockout and WT control mice since frequencies of dividing cells (CFSE^low^) in CD4 and CD8 T cell populations following anti-CD3/CD28 stimulation were similar in the two groups (Fig. 3G). Consistent with the activation and proliferation data, IFN-γ and granzyme B-producing cells derived from CD4 and CD8 populations were not affected by *Slc7a3* depletion (Fig. 3H). These data demonstrate that *Slc7a3* is dispensable for T cell development and activation.

### Slc7a3 deletion does not impact the development of hematological malignancies

**Figure 4:**
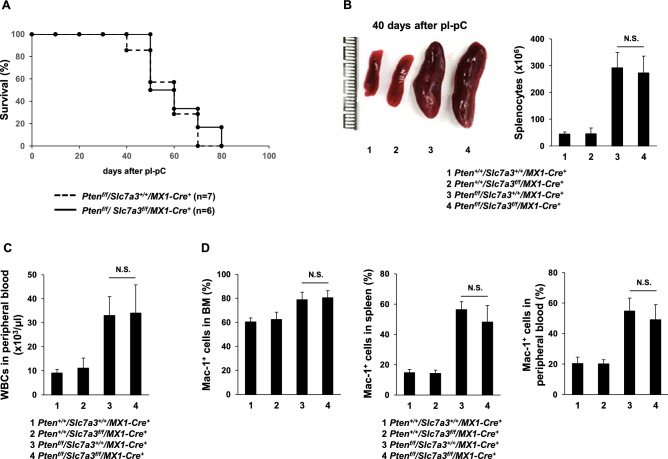
*Slc7a3* knockout does not attenuate *Pten*-loss-evoked leukemogenesis. Six- to eight-week-old *Pten*^*f/f*^*/Slc7a3*^*f/f*^*/MX1-Cre*^+^, *Pten*^*f/f*^*/Slc7a3*^+*/*+^*/MX1-Cre*^+^, *Pten*^+*/*+^*/Slc7a3*^*f/f*^*/MX1-Cre*^+^, and *Pten*^+*/*+^*/Slc7a3*^+*/*+^*/MX1-Cre*^+^ mice were administered pI-pC to induce *Pten* and *Slc7a3* deletion. (**A**) Kaplan–Meier survival curves of *Pten*^*f/f*^*/Slc7a3*^+*/*+^*/MX1-Cre*^+^ (n = 7) and *Pten*^*f/f*^*/Slc7a3*^*f/f*^*/MX1-Cre*^+^ (n = 6) mice. Total numbers of splenocytes (n = 4 mice/genotype) (**B**), WBC counts in the peripheral blood (n = 4 mice/genotype) (**C**), and frequencies of myeloid cells (Mac-1^+^) in the BM, spleen, and PB (D) (n = 4 mice/genotype) were determined six weeks after pI-pC administration.

Arginine is implicated in cancer development and progression^10,12,13^. To determine the possible role of CAT3 (*Slc7a3*)-mediated arginine uptake in tumor development, we assessed the effect of *Slc7a3* deletion on the hematological malignancy driven by *Pten* loss. *Pten*^*f/f*^*/Slc7a3*^*f/f*^*/MX1-Cre*^+^ double knockout mice were generated by crossing *Slc7a3*^*f/*+^*, MX1-Cre*^+^, and *Pten*^*f/*+^^22^ mice. As shown in Fig. 4A, the survival rates of *Pten/Slc7a3* double knockout mice and *Pten* single knockout mice (*Pten*^*f/f*^*/Slc7a3*^+*/*+^*/MX1-Cre*^+^) were indistinguishable. Both *Pten* single and *Pten/Slc7a3* double knockout mice died of hematological malignancies within 80 days. Leukemic burden, as reflected by enlarged spleens (Fig. 4B), increased WBC counts (Fig. 4C), and myeloid leukemic cells (Mac-1^+^) in the BM, spleen, and peripheral blood (PB) (Fig. 4D), was not mitigated in the double knockout mice.

**Figure 5:**
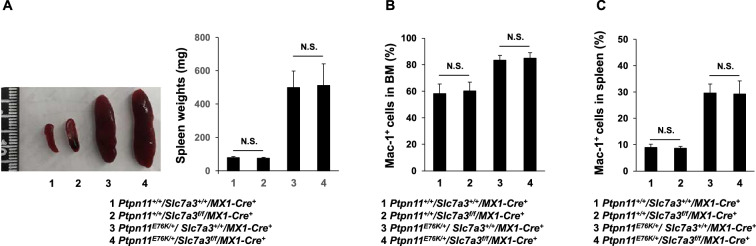
Leukemogenesis driven by the oncogenic *Ptpn11*^*E76K*^ mutation is not affected by *Slc7a3* depletion. Six to eight-week-old *Pten*^*f/f*^*/Slc7a3*^*f/f*^*/MX1-Cre*^+^, *Pten*^*f/f*^*/Slc7a3*^+*/*+^*/MX1-Cre*^+^, *Pten*^+*/*+^*/Slc7a3*^*f/f*^*/MX1-Cre*^+^, and *Pten*^+*/*+^*/Slc7a3*^+*/*+^*/MX1-Cre*^+^ mice were administered pI-pC to induce the oncogenic *Ptpn11*^*E76K*^ mutation and deletion of *Slc7a3*. Spleen weights (normalized against body weights) (**A**), frequencies of myeloid cells (Mac-1^+^) in the BM (**B**) and spleen (**C**) were determined 9 weeks after pI-pC treatment (n = 4 mice/genotype).

Furthermore, we examined the effect of *Slc7a3* depletion on the development of the hematological malignancy induced by a gain-of-function mutation in *Ptpn11* (*Ptpn11*^*E76K/*+^) identified in juvenile myelomonocytic leukemia (JMML)^23^. *Ptpn11*^*E76K/*+^*/Slc7a3*^*f/f*^*/MX1-Cre*^+^ double mutant mice were generated by crossing *Slc7a3*^*f/*+^*, MX1-Cre*^+^, and inducible *Ptpn11*^*E76K*^ mutation knock-in mice (*Ptpn11*^*E76Kneo/*+^)^24,25^. Both *Ptpn11*^*E76K/*+^*/Slc7a3*^*Del*^ double mutant and *Ptpn11*^*E76K/*+^ single mutant mice uniformly developed a JMML-like myeloproliferative neoplasm, as demonstrated by the enlarged spleen (Fig. 5A) and markedly increased myeloid cells (Mac-1^+^) in the BM and spleen (Fig. 5B and C). Altogether, these data suggest that the development and progression of hematopoietic malignancies are not dependent upon *Slc7a3*.

### *Slc7a3* is not required for mouse development

*Slc7a3* has elevated expression levels in embryonic tissues^18^ and is thought to play a major role during embryogenesis and fetal development^14,15^. In addition, *Slc7a3* was found highly expressed in embryonic stem cells (Figure S4). Our qRT-PCR analyses showed that *Slc7a3* levels in mouse embryos at 8.5 days post coitum (dpc) were 5–10 times higher than those in adult tissues (Figure S5A). To determine if *Slc7a3* is important for embryonic development, we generated *Slc7a3* global knockout mice (*Slc7a3*^*f/f*^*/CMV-Cre*^+^) mice by crossing *Slc7a3*^*f/*+^ mice with *CMV-Cre*^+^ mice to delete *Slc7a3* from the germline. These animals were born at the Mendelian ratio without any obvious defects. Their body weights were comparable to those of *Slc7a3*^+*/*+^*/CMV-Cre*^+^ littermates during the 12-months follow-up (Figure S5B). The knockout mice appeared normal, bred well, and had a regular lifespan. Moreover, histopathological examination revealed no abnormalities in adult tissues/organs in these knockout mice (Figure S5C), suggesting that *Slc7a3* is not required for mouse development or maintenance of tissue homeostasis.

### L-arginine uptake is not disrupted by CAT3 (*Slc7a3*) depletion

**Figure 6:**
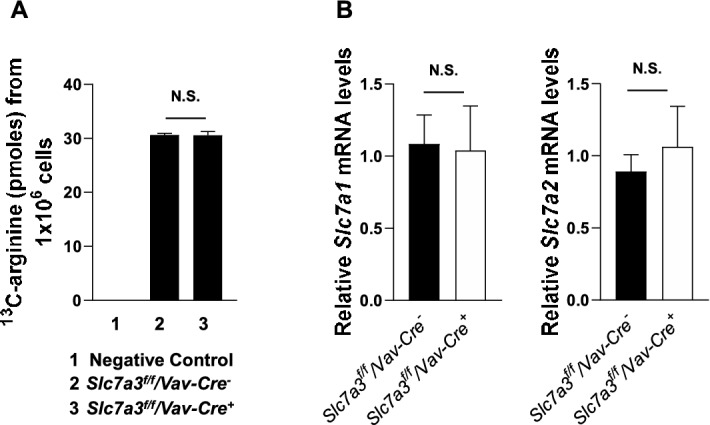
L-arginine uptake is not disrupted in *Slc7a3* knockout cells. (**A**) BM Lin^-^ cells freshly isolated from *Slc7a3*^*f/f*^*/Vav1-Cre*^*−*^ and *Slc7a3*^*f/f*^*/Vav1-Cre*^+^ mice (n = 3 mice/genotype) were starved in arginine-free SILAC RPMI 1640 medium for 2 h. ^13^C labeled L-arginine was added to the culture systems. After 15 min of incubation, cells were collected and washed twice with pre-cooled PBS. Cell pallets (1 × 10^6^ cells/sample) were processed for detection and quantification of ^13^C-arginine by mass spectrometric analyses, as described in Materials and Methods. (**B**) BM Lin^−^ cells were isolated from eight-week-old *Slc7a3*^*f/f*^*/Vav1-Cre*^*−*^ and *Slc7a3*^*f/f*^*/Vav1-Cre*^+^ mice (n = 3 mice/genotype). Total RNA was extracted, and *Slc7a1* and *Slc7a2* mRNA levels were determined by qRT-PCR.

Since no significant defects were detected in either tissue specific or global *Slc7a3* knockout mice, we examined arginine uptake in *Slc7a3* knockout cells with^13^C-labeled L-arginine. Intracellular^13^C-L-arginine was detected and quantified by a Thermo TSQ Quantis mass spectrometer coupled to a Thermo Vanquish UHPLC. To our surprise, *Slc7a3* depleted cells isolated from *Slc7a3*^*f/f*^*/Vav1-Cre*^+^ and WT control littermates had similar arginine uptake efficiency (Fig. 6A), verifying that CAT3 is dispensable for arginine transportation. To determine if other arginine transporters might compensate for the loss of CAT3, we examined the expression of *Slc7a1* (CAT1) and *Slc7a2* (CAT2) in *Slc7a3* knockout BM Lin^−^ cells. No significant differences in *Slc7a1* or *Slc7a2* levels were detected between *Slc7a3* knockout and WT control cells (Fig. 6B).

## Discussion

Amino acids are essential as building blocks of proteins, energy sources, precursors of metabolites, and signaling molecules in the cell. Previous studies have demonstrated the important roles of essential amino acids valine and methionine in HSCs^5,6^. In the present study, we have unexpectedly found that exogenous arginine, a semi-essential amino acid, is required for the maintenance and/or function of ex vivo cultured HSCs. Notably, the detrimental effect of arginine deprivation on HSCs was even greater than that caused by the starvation of essential amino acids such as valine^5^, and other important amino acids such as methionine^6^. The underlying mechanism is unclear. The important role of exogenous arginine for HSCs is also supported by the observation that *Slc7a3* (CAT3), a major arginine transporter, was upregulated by ~ 17-fold in hematopoietic stem/progenitor cells that had defective mitochondrial metabolism^20^. Surprisingly, this transporter is dispensable for HSC maintenance and function. Hematopoietic cell development in steady-state or in response to hematopoietic stress was not affected in *Slc7a3* knockout mice. Moreover, although *Slc7a3* is highly expressed in naïve and activated CD8 T cells, neither T cell development nor activation/proliferation was impacted by *Slc7a3* depletion. Arginine uptake assays revealed that arginine transportation was not disrupted in *Slc7a3* knockout cells.

Selective arginine depletion with recombinant arginine-degrading enzymes and arginine transportation inhibition has been exploited or proposed as a therapeutic strategy for the treatment of cancers due to the important role of arginine-mediated metabolism and signaling in cancer cells^8–13^. Our data presented in this report does not suggest that the arginine transporter CAT3 is a useful therapeutic target for controlling cancer cell access to arginine because *Pten* loss or an oncogenic *Ptpn11* mutation-driven leukemia development is not impacted by *Slc7a3* depletion. Arginine can be accumulated by a variety of amino acid transporter systems^14,15^. Three cationic amino acid transporters (CATs) are relatively selective for arginine but differ in their substrate affinities and sensitivities to trans-stimulation. Different CATs have distinct distribution patterns in various cell types. Their expression is highly regulatable at the levels of transcription, mRNA stability, translation, and subcellular localization^14,15^. *Slc7a1* (CAT1) and *Slc7a2* (CAT2) expression levels were not significantly changed in *Slc7a3* (CAT3) knockout cells. It is possible that CAT1 and CAT2 were functionally upregulated to compensate arginine transportation in the absence of CAT3. Other Na^+^-independent cationic amino acid transport systems (system y^+^) or heteromeric amino acid transporters could also compensate for the loss of CAT3 function. The specific amino acid transporter(s) that conduct(s) arginine transportation after *Slc7a3* depletion remain(s) to be determined.

## Materials and methods

### Mouse studies

*Slc7a3*^*f/*+^ mice were generated in this study by gene-targeting, in which Exon 4–9 were flanked by two *Loxp* sites through homologous recombination (Fig. S1A). Conditional *Ptpn11*^*E76K*^ mutation knock-in mice (*Ptpn11*^*E76K-neo/*+^) were generated in our previous study^24^. *MX1-Cre*^+^, *CMV-Cre*^+^, and *Pten*^*f/*+^ mice in the C57BL6 background were obtained from The Jackson Laboratory. *Vav1-Cre*^+^ mice were provided by Dr. Thomas Graf at Center for Genomic Regulation and ICREA, Spain. These mice were intercrossed to produce mice of various genotypes. Mice were kept under specific pathogen-free conditions in Division of Animal Resources, Emory University. Mice of the same age, sex, and genotype were randomly grouped for subsequent experiments (investigators were not blinded). All animal procedures complied with the NIH Guidelines for the Care and Use of Laboratory Animals and were approved by the Institutional Animal Care and Use Committee.

### In-vitro culture of LSK cells in the medium lacking single amino acids

Sorted HSCs-enriched LSK cells (Lin^-^Sca-1^+^c-Kit^+^) were seeded in 96-well plates at the concentration of 500 cells/200 µl. They were cultured in Earle’s Balanced Salt Solution supplemented with 10% FBS, MEM vitamins (D-biotin, choline chloride, folic acid, *myo*-inositol, niacinamide, D-pantothenic acid, pyridoxal-HCL, riboflavin, thiamine-HCL, vitamin B12) (1x), SCF (50 ng/ml), IL-3 (20 ng/ml), IL-6 (20 ng/ml), and a mixture of amino acids that lacked a specific amino acid. The final concentration of alanine, arginine, asparagine, aspartic acid, cysteine, glutamic acid, glutamine, glycine, histidine, isoleucine, leucine, lysine, methionine, phenylalanine, proline, serine, threonine, tryptophan, tyrosine, and valine in the medium was 0.025, 0.084, 0.028, 0.030, 0.092, 0.075, 0.584, 0.03, 0.042, 0.105, 0.105, 0.146, 0.03, 0.066, 0.04, 0.042, 0.095, 0.016, 0.104, and 0.094 mg/ml, respectively. Seven days later, cells in each well were photographed.

### Flow cytometry analyses and cell sorting

BM cells freshly collected from bilateral hindlegs were stained with antibodies labeled with various fluorochromes: PE-labeled antibodies against lineage markers (Mac-1, Gr-1, Ter119, B220, CD3e), Sca-1-PE-Cy7, c-Kit-APC-eFluor 780, CD150-Alexa Fluor 647, and CD48- PerCP-eFluor710. Specific cell populations, Lin^+^ cells, LK cells (Lin^−^Sca-1^-^c-Kit^+^), LSK cells (Lin^−^Sca-1^+^c-Kit^+^), and HSCs (Lin^−^Sca-1^+^c-Kit^+^CD150^+^CD48^−^) were identified and gated based on immunophenotypes for quantification. Similarly, freshly harvested BM cells were stained with PE-labeled lineage antibodies as above, Sca-1-PE-Cy7, c-Kit-APC-eFluor 780, CD34-Alexa Fluor 647, CD16/32-FITC, and CD127-PE-Cy5. The proportions of lineage progenitors CMPs (Lin^-^Sca-1^−^c-Kit^+^CD16/32^med/low^ CD34^+^), GMPs (Lin^−^Sca-1^−^c-Kit^+^CD16/32^high^CD34^+^), MEPs (Lin^−^Sca-1^−^c-Kit^+^CD16/32^med/low^CD34^−^), and CLPs (Lin^−^Sca-1^low^c-Kit^low^CD127^+^) were determined by multiparameter FACS. For analyses of lineage development in BM, fresh BM cells were stained with the following antibodies: Mac-1-PE, B220-APC, CD3-APC-eFluor 780, CD4-PE-Cy7, CD8-BV605. Frequencies of specific lineage cells were quantified by FACS. Hematological malignancies induced by the loss of *Pten* or the gain-of-function mutation of *Ptpn11* (*Ptpn11*^*E76K/*+^) were examined by FACS analyses. In brief, BM cells, splenocytes, and peripheral blood cells were collected and stained with Mac-1 antibody-PE. Percentages of Mac-1^+^ myeloid cells were determined by FACS. For T cell analyses, thymocytes freshly collected from 6 to 8-week-old *Slc7a3*^*f/f*^*/Vav1-Cre*^+^ mice and *Slc7a3*^*f/f*^*/Vav1-Cre*^*−*^ littermates were stained with CD4-PE-Cy7, CD8-APC, CD44-FITC, and CD25-PE antibodies. Frequencies of CD4^+^, CD8^+^, DP (double-positive, CD4^+^CD8^+^), DN1 (double-negative 1, CD4^−^CD8^−^CD44^+^CD25^−^), DN2 (CD4^−^CD8^−^CD44^+^CD25^+^), DN3 (CD4^−^CD8^−^CD44^−^CD25^+^), and DN4 (CD4^−^CD8^−^CD44^−^CD25^−^) cells were quantified. In addition, splenocytes were collected and stained with the following antibodies: Ter119-FITC, CD3-APC- eFluor 780, CD4-PE-Cy7, CD8-PE, CD44-APC, and CD62L-Pacific Blue. Percentages of naïve T cells (CD44^−^CD62L^+^), central memory T cells (T_CM_, CD44^+^CD62L^+^), and effector memory T cells (T_EM_, CD44^+^CD62L^−^) in CD4^+^ or CD8^+^ T cell populations were quantified by FACS.

For LSK cell sorting, BM cells were lineage-depleted using a Lineage Cell depletion kit, followed by staining with fluorochrome-labeled antibodies: PE-labeled antibodies against lineage markers (Mac-1, Gr-1, Ter119, B220, CD3e), Sca-1-PE-Cy7, c-Kit-APC-eFluor 780 antibodies. The cells were processed for sorting using BD FACS ARIA II.

### Colony-forming unit assay

Freshly harvested BM cells (2 × 10^4^ cells/ml) were seeded in 0.9% methylcellulose IMDM medium containing 15% fetal bovine serum (FBS), glutamine (10^–4^ M), β-mercaptoethanol (3.3 × 10^–5^ M), SCF (50 ng/ml), IL-3 (20 ng/ml), IL-6 (20 ng/ml), and erythropoietin (EPO, 3 units/ml). After 10 days of culture at 37 °C in a humidified 5% CO_2_ incubator, hematopoietic colonies (CFU-GEMM, CFU-GM, and BFU-E) consisting of more than 50 cells were counted under an inverted microscope. The mean value of the triplicates from each mouse was used for final statistical analyses.

### T cell activation and proliferation assay

Splenocytes freshly isolated from 6 to 8-week-old *Slc7a3*^*f/f*^*/Vav1-Cre*^+^ mice and control littermates were cultured in RPMI1640 medium supplemented with 10% FBS, 1% P/S, and hIL-2 (30 unit/ml) (1 × 10^6^ cells/200 µl per well). These cells were stimulated with T-Activator Dynabeads (anti-CD3/CD28 antibodies) (2 µl/10^6^ cells) for 24 h. The cells were collected and stained with CD4-PE-Cy7, CD8-BV605, CD69-APC/Cyanine7, and CD25-PE antibodies. Percentages of early activated T cell populations (CD25^+^ or CD69^+^) in CD4^+^ and CD8^+^ T cell populations were determined by FACS. For proliferation assays, freshly harvested splenocytes were loaded with CFSE (5 nM) for 10 min in a 37 °C 5% CO_2_ incubator and washed three times with PBS before being seeded into culture plates (1 × 10^6^ cells/200 µl). The cells were activated with T-Activator Dynabeads as described above and further cultured for 4 days. They were then collected and stained with CD4-PE-Cy7 and CD8-APC antibodies. Percentages of the cells with lower CFSE intensities were quantified. In addition, the harvested splenocytes were stimulated and cultured for four days as above and then treated with Cell Activation Cocktail (2 μL for each ml of cell suspension) and Golgistop (4 µl for every 6 ml of culture) for 4 h. The cells were collected and stained with CD4-PE-Cy7 and CD8-BV605 antibodies. They were then fixed and permeabilized by using a Cytofix/Cytoperm kit and stained with IFN-γ-APC, and Granzyme B-PE antibodies. Percentages of IFN-γ^+^ and Granzyme B^+^ cells were quantified by FACS.

### L-arginine uptake assay

Lineage-depleted (Lin^−^) cells were isolated from BM cells freshly harvested from 6-week-old *Slc7a3*^*f/f*^*/Vav1-Cre*^+^ mice and control littermates (*Slc7a3*^*f/f*^*/Vav1-Cre*^*−*^) by a Lineage Cell Depletion Kit. Lin^−^ cells (1 × 10^6^) were starved in arginine-free SILAC RPMI 1640 medium for 2 h. ^13^C labeled L-arginine (0.5 mM) was then added to the culture systems. After 15 min of incubation, cells were collected, washed twice with pre-cooled PBS, and snap-frozen at − 80 °C. Cell pallets (1 × 10^6^ cells/sample) were processed for detection and quantification of ^13^C-arginine by a Thermo TSQ Quantis mass spectrometer coupled to a Thermo Vanquish UHPLC.

### Ethics approval

All animal experimental procedures complied with the NIH Guidelines for the Care and Use of Laboratory Animals, followed the recommendations in the ARRIVE guidelines, and were approved by the Institutional Animal Care and Use Committee of Emory University.

## Quantification and statistical analysis

Data are presented as mean ± SD of biological replicates. Unless otherwise specified, statistical analyses were performed using two-tailed Student’s *t*-test for the majority of comparisons. Comparisons of overall survival rates were performed using the Mantel_Cox log-rank test. *p < 0.05; **p < 0.01; ***p < 0.001. N.S., not significant.

## Supplementary Information

Supplementary Information.

## Data Availability

The original data that support the findings of this study and the experimental protocols are available to interested investigators from the authors on reasonable request, see author contributions for specific data sets. Requests for the data from this study should be directed to Cheng-Kui Qu at cheng-kui.qu@emory.edu.
