# Supplementary material for: Extracellular arginine is required but the arginine transporter CAT3 (Slc7a3) is dispensable for mouse normal and malignant hematopoiesis
Source: Sci Rep. 2022 Dec 17;12:21832. doi: 10.1038/s41598-022-24554-2 (PMC9759514; doi:10.1038/s41598-022-24554-2)
Supplement: Supplementary file 1 — Supplementary Information. [file 41598_2022_24554_MOESM1_ESM.pdf]

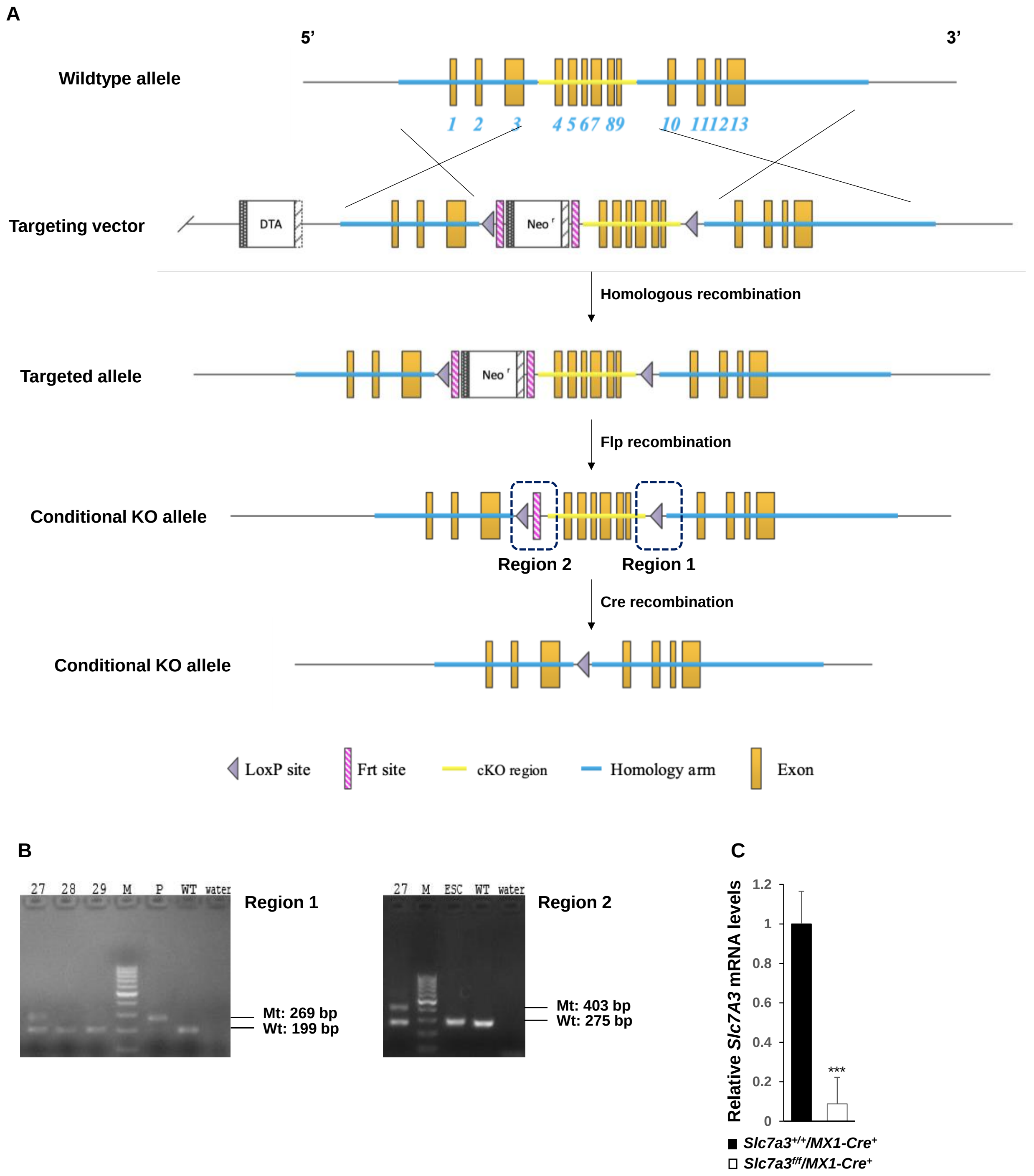
A
5'
3'
Wildtype allele
1 2 3 4 5 6 7 8 9 10 11 12 13
DTA
Neo r
Targeting vector
Homologous recombination
Targeted allele
Flp recombination
Conditional KO allele
Region 2
Region 1
Cre recombination
Conditional KO allele
LoxP site
Frt site
cKO region
Homology arm
Exon
B
27 28 29 M P WT water
Region 1
Mt: 269 bp
Wt: 199 bp
27 M ESC WT water
Region 2
Mt: 403 bp
Wt: 275 bp
C
Relative Slc7A3 mRNA levels
1.2
1
0.8
0.6
0.4
0.2
0
***
Slc7a3+/+/MX1-Cre+
Slc7a3f/f/MX1-Cre+

Supplementary Figure S1. Generation and characterization of *Slc7a3* conditional knock-out mice. (A) The gene targeting strategy for generation of a conditional *Slc7a3* allele. (B) Correctly targeted embryonic stem (ES) cell clones were identified by Southern blotting. Two independent ES cell clones were selected for blastocyst microinjections to generate chimeric mice. Germline-transmitted chimeric mice were used to crossbreed with *Flp* deleter mice to delete the *Frt* flanked *neo* cassette. *Slc7a3*$^{f/+}$ (Mt) mice were identified by PCR genotyping of genomic DNA for Region 1 and the positive mice were confirmed by PCR genotyping of genomic DNA for Region 2. Wt, wild-type genomic DNA. (C) Eight-week-old *Slc7a3*$^{f/f}$/*MX1-Cre*$^{+}$ and *Slc7a3*$^{+/+}$/*MX1-Cre*$^{+}$ mice were administered 3 doses of pI-pC (1.0 µg/g body weight, i.p.) every other day over 5 days. Five weeks after pI-pC administration, total RNA was extracted from BM cells. *Slc7a3* mRNA levels were determined by qRT-PCR (n = 4 mice/genotype).

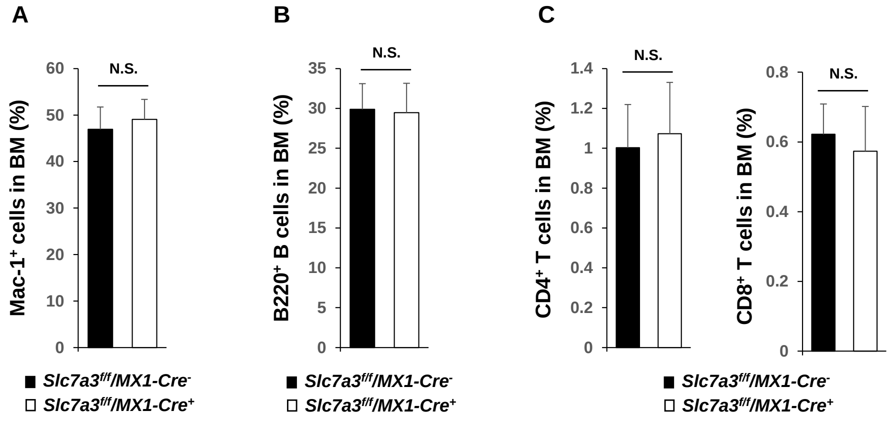

Supplementary Figure S2. Deletion of *Slc7a3* has no effect on blood cell development. Six-week-old *Slc7a3$^{f/f}$/MX1-Cre*$^{+}$ mice and *Slc7a3$^{+/+}$/MX1-Cre*$^{+}$ littermates were administered pI-pC (n = 5 mice/genotype). Eight weeks following pI-pC administration, mice were sacrificed, and BM cells were processed for FACS analyses to quantify percentages of myeloid cells (Mac-1^{+}) (A), B lymphoid cells (B220^{+}) (B), and T lymphoid cells (CD4^{+} and CD8^{+}) (C).

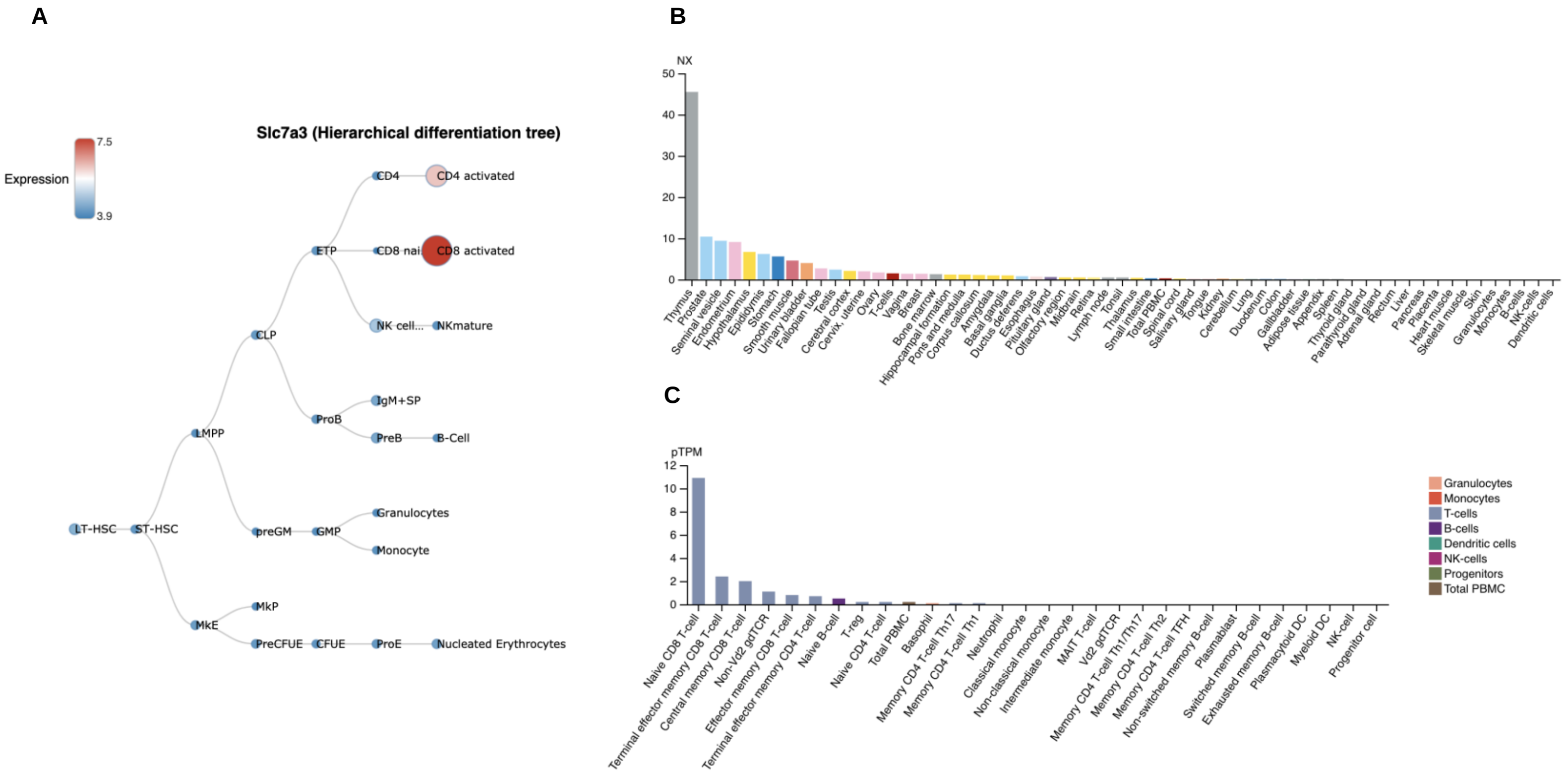

Supplementary Figure S3. Expression pattern of *Slc7a3* in blood cell populations and other tissues. (A) *Slc7a3* mRNA levels in mouse normal hematopoietic cells at different maturation stages based on the database Bloodspot (https://servers.binf.ku.dk/bloodspot). (B) *Slc7a3* RNA levels in human tissues based on the Consensus dataset in Human Protein Atlas (https://www.proteinatlas.org). NX, Normalized Expression. Color-coding is based on tissue groups, each consisting of tissues with functional features in common. (C) *Slc7a3* mRNA levels in human normal peripheral blood mononuclear cells (PBMCs) from healthy donors, including 29 immune cell types within PBMCs based on Monaco Scaled dataset in Human Protein Atlas (https://www.proteinatlas.org). pTPM, transcripts per million protein-coding genes.

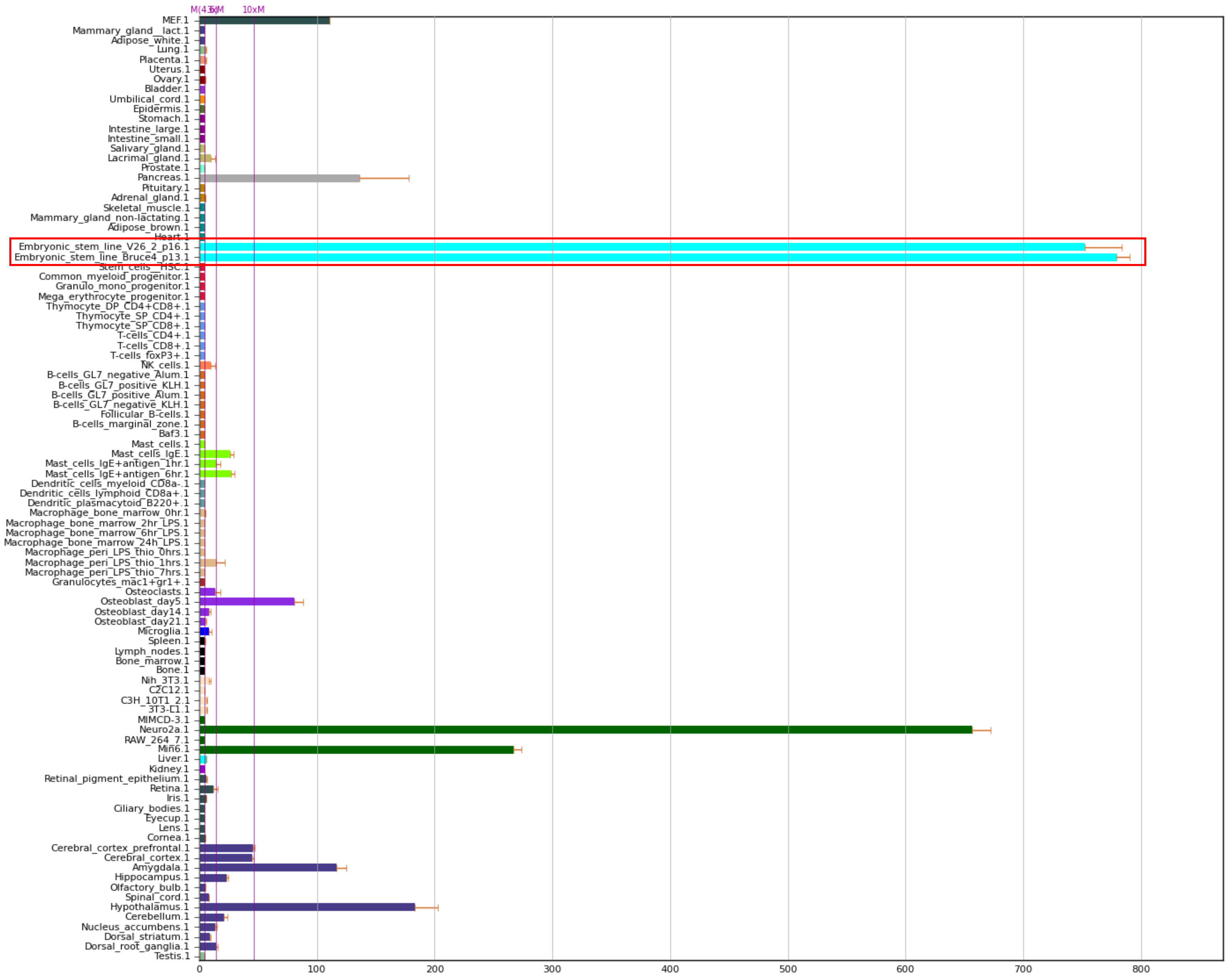

Supplementary Figure S4. *Slc7a3* is highly expressed in embryonic stem cells. *Slc7a3* mRNA expression levels in a diverse array of normal mouse tissues, organs, and cell lines based on the dataset in BioGPS (http://biogps.org). Color-coding is based on tissue groups, each consisting of tissues with functional features in common. *Slc7a3* expression levels in embryonic stem cells are highlighted in a red frame.

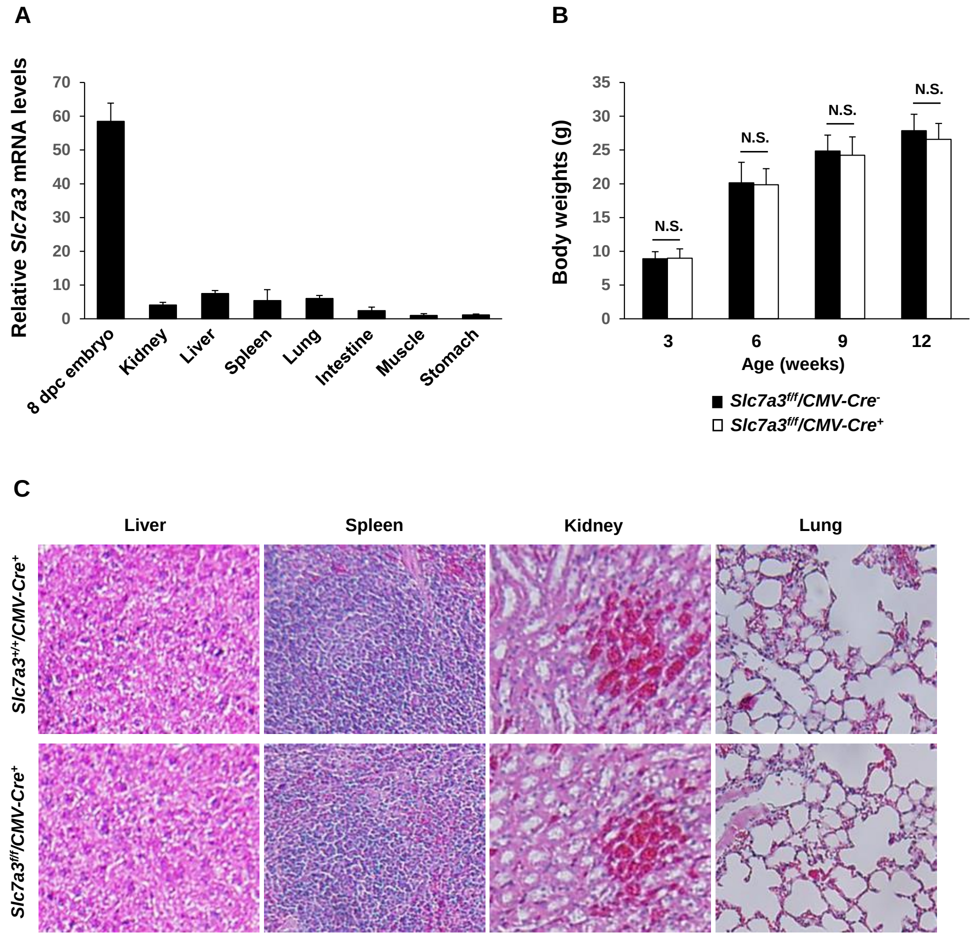

Supplementary Figure S5. Global deletion of *Slc7a3* does not impact mouse development. (A) Total RNA was extracted from the indicated tissues isolated from healthy WT C57BL/6 mice (n=3 mice). *Slc7a3* mRNA levels in these tissues were determined by qRT-PCR. (B) Body weights of *Slc7a3*$^{+/+}$*/CMV-Cre*$^{+}$ mice and *Slc7a3*$^{f/f}$*/CMV-Cre*$^{+}$ littermates were measured at the indicated ages (n=3 mice/genotype). (C) Liver, spleen, kidney, and lung tissues isolated from six-month-old *Slc7a3*$^{+/+}$*/CMV-Cre*$^{+}$ mice and *Slc7a3*$^{f/f}$*/CMV-Cre*$^{+}$ littermates were processed for histopathological examination (hematoxylin and eosin staining) (n=3 mice/genotype). Representative pictures are shown.
